# The Landscape of Small Leucine-Rich Proteoglycan Impact on Cancer Pathogenesis with a Focus on Biglycan and Lumican

**DOI:** 10.3390/cancers15143549

**Published:** 2023-07-09

**Authors:** Aikaterini Berdiaki, Eirini-Maria Giatagana, George Tzanakakis, Dragana Nikitovic

**Affiliations:** Laboratory of Histology-Embryology, Medical School, University of Crete, 71003 Heraklion, Greece; berdiaki@uoc.gr (A.B.); eirini_gt@hotmail.com (E.-M.G.); tzanakak@uoc.gr (G.T.)

**Keywords:** small-leucine rich proteoglycans (SLRPs), cancer, biglycan, lumican, extracelllar matrix, cancer invasion, cancer-associated inflammation

## Abstract

**Simple Summary:**

Cancer is a complex disease in which both cells and their environment are altered. A tumor microenvironment contains tumor cells, normal tissue cells, blood vessels, cells of the immune system, stromal cells, and the extracellular matrix (ECM). The small leucine-rich proteoglycans (SLRPs) are molecules that consist of a protein core and glycosaminoglycan chains. SLRPs are released by the cells into the surrounding matrix. These biomolecules can react with molecules on the cell surface and secreted biomolecules and modify signaling, which regulates cell behavior. Their expression changes during cancer development, contributing to cancer growth and metastases. This review will focus on the roles of two SLRP members—biglycan and lumican—which are shown to affect cancer cells’ survival, growth, and spread. We will also discuss their mechanisms of action and possible therapeutic uses.

**Abstract:**

Cancer development is a multifactorial procedure that involves changes in the cell microenvironment and specific modulations in cell functions. A tumor microenvironment contains tumor cells, non-malignant cells, blood vessels, cells of the immune system, stromal cells, and the extracellular matrix (ECM). The small leucine-rich proteoglycans (SLRPs) are a family of nineteen proteoglycans, which are ubiquitously expressed among mammalian tissues and especially abundant in the ECM. SLRPs are divided into five canonical classes (classes I–III, containing fourteen members) and non-canonical classes (classes IV–V, including five members) based on their amino-acid structural sequence, chromosomal organization, and functional properties. Variations in both the protein core structure and glycosylation status lead to SLRP-specific interactions with cell membrane receptors, cytokines, growth factors, and structural ECM molecules. SLRPs have been implicated in the regulation of cancer growth, motility, and invasion, as well as in cancer-associated inflammation and autophagy, highlighting their crucial role in the processes of carcinogenesis. Except for the class I SLRP decorin, to which an anti-tumorigenic role has been attributed, other SLPRs’ roles have not been fully clarified. This review will focus on the functions of the class I and II SLRP members biglycan and lumican, which are correlated to various aspects of cancer development.

## 1. Introduction

Cancer development is a multifactorial procedure that involves changes in the cell microenvironment and specific modulations in cell functions. Essential cancer cell functions, including growth, adhesion, migration, and survival, are affected by genetic, metabolic, and environmental factors [1,2].

A tumor microenvironment (TM) contains tumor cells, non-malignant cells, blood vessels, cells of the immune system, stromal cells, and the extracellular matrix (ECM) [3,4]. Interactions between the microenvironment components are established to determine cancer progress [5]. ECM constituent expression is modulated in cancer and between different cancer types, resulting in the modulation of cell function and cancer development [6,7]. Indeed, the alterations of specific ECM components [8,9,10] have been suggested as disease progression markers.

The complex ECM net consists of fibrillar proteins, such as collagen and elastin, as well as proteoglycans (PGs), glycoproteins, and glycosaminoglycans (GAGs) that have the ability to regulate tissue homeostasis [11]. Notably, changes in the ECM component of the TM enhance tumor cell functions, such as proliferation, cellular energetics, invasion, and resistance to apoptosis, while supporting tumor angiogenesis, thus inhibiting immune cell action and tumor immune invasion [12,13].

The small leucine-rich proteoglycans (SLRPs) are ubiquitously expressed among mammalian tissues and are especially abundant in the ECM [14]. The nineteen SLRP members are classified into five distinguishable classes based on their amino-acid structural sequence, chromosomal organization, and functional properties. The five classes are defined as canonical (Classes I–III, including fourteen members) and non-canonical classes (Classes IV–V, including five members) [15].

SLRPs interact with structural ECM molecules, cell membrane receptors, growth factors, and cytokines, as well as with their protein cores and GAG chains [16,17]. Notably, GAG chains can modify protein core–ligand interactions by introducing immense variability in their signaling [18,19,20]. A plethora of studies have demonstrated that SLRPs’ expression is altered in tumors [21,22] and that by interacting with tyrosine kinase receptors or growth factors, they affect tumor behavior [23,24,25,26]. Moreover, in the case of tissue insult and damage, SLRPs bind toll-like receptors (TLRs) and are released to act as damage-associated molecular patterns [27]. These versatile biomodulators also affect cancer-associated inflammation and autophagy [28,29]. Except for the class I SLRP decorin, to which an anti-tumorigenic role has been attributed, other SLPRs’ functions still need to be fully clarified.

This review will focus on the roles of class I and II SLRP members biglycan and lumican, which have been correlated to various aspects of cancer development.

## 2. The Tumor ECM

The ECM macromolecules have configurational and physicochemical properties that discretely support the realization of their tissue-specific physiological roles [30]. As demonstrated in keratinocyte-derived tumors, the changed mechanics of the pre-malignant and malignant lesions promote cancer progression [31]. In healthy tissues, collagen fibers primarily define mechanical strength and tissue stiffness, whereas PGs/GAGs, by bridging collagen fibrils, regulate the compressive rigidity of tissues and ECM viscoelasticity. Modifying the interactions, density, and crosslinking of ECM fibers induces irregular matrix stiffness that is correlated to the transformation of pre-malignant lesions into cancer [32]. Thus, aberrant collagen accumulation in the tumor ECM, due to the action of cancer-associated fibroblasts (CAFs), results in the production of dense ECM, leading to fibrosis and, in the case of breast tissue, enhanced mammographic density that facilitates malignant transformation [33,34].

Another critical component of the ECM are PGs, which are made up of a protein core covalently linked to one or more GAG chains. PGs can be decorated via the following discrete GAG chains: heparan sulfate (HS), chondroitin sulfate (CS), dermatan sulfate (DS), and keratan sulfate (KS). These pleiotropic biomolecules are either secreted into the ECM or connected with the cell membranes, but can also be localized intracellularly in the case of serglycin. After being secreted into the ECM, PGs can be deposited “away” from the cell, bind into matrix fibers, form a part of the pericellular supermatrix complexes, or be incorporated into basement membranes [17]. Studies examining the variabilities in the protein core and glycosylation patterns have described 45 different PGs [16]. However, the localization of secreted and cell membrane PGs can be changed due to alterations in synthesis/endocytosis or shedding [25,35]. The expression of PGs, their ECM, and cellular and specific subcellular localization varies between cancer types, thus modulating cell function and tumor differentiation [1,6]. For example, epithelial tumors exhibit a different PG phenotype than that of mesenchymal tumors [36]. Furthermore, PGs contribute to the mechanisms of cancer-associated modulation of the immune response [1].

## 3. SLRPs Structure and Roles

The SLRP family members harbor a variable number of discrete leucine-rich repeats (LRR) in their relatively small protein cores (40 to 60 kDa). Notably, the LRRs motifs display different amino acid sequences across the SLRP family (size range 20–29 residues), which typically contain a conserved hydrophobic motif wherein leucine residues can be exchanged by separate hydrophobic amino acids [37]. The N- and C-terminal regions of the SLRPs’ protein cores have abundant cysteine residues [16,38,39]. The most extended LRR tandem domain, which protrudes laterally from the core protein, is known as the ear-repeat and consists of the penultimate C-terminal LRRs [16,38]. SLRP structure and ligand binding are modulated via the residue sequences of the LRRs, the glycanation pattern, the number of cysteine residues, and the ear-repeats, as well as the extent of tyrosine-residue sulfation [15].

SLRPs’ concave faces are made up of β-sheets, whereas α-helices create their convex surfaces. Furthermore, each LRR has α-helix and one β-sheet that correlate to a one-turn tube-like structure [40]. The resulting structure mediates protein–protein interaction with the inner concave face and facilitates ligand attachment.

Post-translational glycosylation differs between SLRP family members. It must be pointed out that some SLRPs are not typical PGs [41,42]. For example, decorin and biglycan, members of the canonical class I bear CS/DS side chains, whereas the asporin member does not bear these chains [38,43]. Lumican, which is a class II member, is decorated with KS chains or polylactosamine bound into LRRs [44]. However, class III members can, likewise, be glycanated with KS (osteoglycin), CS/DS (epiphycan), or no (opticin) GAG chains [41,45]. Nevertheless, most non-canonical classes IV and V members do not bear GAG chains, except for chondroadherin, which is glycanated with KS [16,46].

Variation in the glycosylation status leads to SLRP-specific interactions with cell membrane receptors, cytokines, growth factors, and structural ECM molecules [18]. Thus, the biglycan with two GAG chains binds collagen I much more efficiently than the biglycan protein core or the class I SLRP decorin [47,48]. However, interestingly, the SLPs’ glycanation statuses can create the basis of a chronological age “footprint” by acting as an intervertebral disc and articular cartilage. The expression of biglycan GAG-free form is low in adolescents, enhances with age, and is predominantly expressed in adults [49,50]. Recently, biglycan, decorin, fibromodulin, and lumican were shown to discretely inhibit MMP-14 activity through specific binding, which was affected by their glycosylation pattern [51].

In summary, SLRPs’ actions are mediated through their core protein and GAG chains [44,52,53]. For example, their core proteins associate with collagen fibers, whereas their GAG chains determine the diameter of collagen fibers, their intermolecular crosslinking, and their ability to aggregate into thicker bundles [47,54], enabling tissue scaffold formation [16]. Furthermore, SLRPs modulate the access of collagenases to their cleavage sites, regulating collagen proteolysis and, in extrapolation, ECM composition [55]. Finally, as discussed in bone tissue, SLRPs, by binding a plethora of cell membrane receptors, growth factors, and cytokines, can initiate and regulate specific intracellular signaling, thereby modulating cell functions [53]. Notably, a multitude of studies have shown that SLRPs’ interaction with tyrosine kinase receptors or growth factors regulates biological functions and tumor progression [23,24,25,26]. In addition, SLRPs can be secreted upon tissue insult, bind toll-like receptors (TLRs), and take on the role of damage-associated molecular patterns [27]. Furthermore, SLRPs have been implicated in the regulation of cancer-associated inflammation and autophagy, highlighting their crucial role in the processes of carcinogenesis [28].

### 3.1. Biglycan Structure

Biglycan is a class I SLRP with a core protein molecular weight 42-kDa. Its gene is located at the Xq28 position [56], and its protein core consists of 10 to 12 LRRs, which are flanked by cysteine-rich regions. Like all class I SLRPs, the biglycan gene contains eight exons in which the intron/exon junctions are highly conserved. Biglycans’ structures are thoroughly described and discussed by Schaefer and Iozzo (2008) and Schaefer and Schaefer (2010) [17,41]. In addition, recent studies identified specific interactions of the biglycan protein core with biological mediators. Thus, the LRR2-3 peptide’s structural and charge-bearing properties support the interaction with bone morphogenetic protein-2 (BMP-2) and its partner receptors, facilitating BMP-2 osteogenic function [57].

Human biglycan has been defined as containing 1–2 GAG chains bound at the N-terminus amino acids 5 and 10 [58]. CS or DS chains can be associated with the protein core in a tissue-specific manner [58]. Furthermore, the non-glycated biglycan has been associated with the aging articular cartilage and is a product of proteolysis of the amino-terminal region [50]. Alterations in biglycan’s glycanation status have been correlated to various pathologies. Thus, aberrant proteoglycan glycosylation results in disturbed osteoblast differentiation, forming the basis for bone fragility in a gerodermia osteodysplastica murine model [19]. Recently, precise modulation in the biglycan CS/DS chain has been identified. Thus, bisulfated saturated and trisulfated unsaturated disaccharides were detected. IMS MS/MS analysis demonstrated that at the CS/DS chains, non-reducing end 3-O-sulfated GlcA harbors a rare bisulfated motif that is possibly relevant to cancer-associated signaling [20]. In addition, biglycan can bear N-linked oligosaccharide chains bound into its central LRRs motifs, which are able to specifically modify biglycan-binding abilities [59].

### 3.2. Lumican Structure

Lumican is a class II SLRP with a core protein molecular weight of 38-kDa, which exhibits four distinguishable regions. Specifically, these regions encompass (i) a peptide composed of 16 amino acids, (ii) an N-terminal region a bearing negative charge and containing disulfide bonds and tyrosine sulfate, (iii) a region containing 6–10 LRR motifs capable of supporting protein interactions, and (iv) a C-terminal region harboring two conserved cysteine residues [39]. Lumican was described as a crucial PG of the chick cornea [60,61]. The analysis of the lumican amino acid sequence discovered four putative positions of KS chain or oligosaccharide substitution in the LRR region [62]. However, later data indicated that not all putative positions were available for KS substitution [63].

In addition to its KS decoration, lumican may be substituted for polylactosamines of different sizes; alternatively, it can be in non-glycosylated form [64]. The non-glycanated lumican form is shown to increase with age due to decreased KS synthesis [65].

The gene of this class II SLRP is located on chromosome 12q21.3–q22 [62]. Notably, the lumican expression of discrete tissues changes drastically in a development stage-dependent manner. Thus, an early expression of lumican in chick cornea during fetal development is evident [66], whereas human cartilage expresses lumican after birth [67]. These data suggest that lumican exhibits species-specific roles during fetal development. Unsurprisingly, lumican exerts critical functions in tissue organization, which are highlighted by a study showing that lumican deficiency induces the formation of collagen fibrils with increased diameters and aberrant ECM matrices [68].

## 4. Biglycan and Lumican Expression and Roles in Cancerogenesis

Under homeostasis, the majority of secreted biglycan is bound to matrix fibers. The biglycan that is attached to an intercellular matrix is cleaved by proteases and released under pathological conditions [21,69]. In TME, both cancer and stroma cells can overexpress biglycan [21]. Furthermore, it can also be de novo released by activated macrophages [70]. A critical role in regulating cancer progression has been attributed to biglycan, though this role must be fully clarified. Thus, overexpression of biglycan mRNA has been detected in the vast majority of cancer tissues, including breast, bladder, gastric, colorectal, central nervous system, lung, head, neck, esophageal, and ovarian cancer tissues, as well as another twenty-eight cancer types, using the Oncomine database [22].

Furthermore, biglycan protein is determined to be overexpressed in human endometrial cancer, both regarding the parenchyma and the mesenchyme compartments [71]. Notably, its expression in the cancerous, mesenchyme-derived stroma was positively associated with poor prognosis [71]. In addition, biglycan protein and mRNA expression were reported to be elevated in gastric cancer tissues compared to adjacent healthy tissue, whereas its gene expression was correlated to gastric cancer metastases [72]. Likewise, higher expression of biglycan was correlated with shorter overall survival in gastric cancer patients [73].

Analysis of a cohort of oral squamous cell carcinoma biopsies revealed that patients with higher biglycan and lower decorin expression exhibited poor overall survival, as well as tumor-specific survival [74].

A database study evaluating the function and protein–protein interaction network of coexpression genes showed that biglycan facilitates the incidence of lung adenocarcinoma through ECM–receptor interaction [75]. Moreover, an association between biglycan, mRNA, and protein expression was also identified in bladder, esophagus, and colorectal cancer [76,77,78,79]. Biglycan was recognized as one of the signature genes utilized as a metastasis-specific biomarker in human colon cancer using integrated analysis [80]. In addition, overexpressed biglycan in prostate cancer tissues was strongly correlated with disease development [81]. Recently, in a retrospective study, biglycan expression in the cancerous prostate stroma was suggested to be a potential prognostic factor for this cancer [82]. Considering the high incidence of prostate cancer in older men, this finding could have significant treatment outcomes [83]. However, prospective studies are needed to confirm the validity of biglycan as an independent prospective marker for prostate cancer and other cancer types.

The majority of studies seem to indicate that high biglycan expression is a negative factor in cancer development and progression. Recently, a study focusing on pan-cancer tumor bulks and the number of single-cell RNA sequencing cohorts demonstrated increased biglycan secretion by CAFs [84]. Secreted biglycan was negatively correlated with patient survival and response to therapy [84]. These biglycan effects could be attributed to its known immunomodulatory role [69,85].

In contrast, a cohort study on bladder cancer patients showed that high biglycan expression was associated with reduced tumor cell growth and enhanced ten-year survival in patients [86]. Also, a recent study stated that biglycan protein expression is reduced in breast cancer tissue compared to normal tissue [87]. These authors utilized a deep-learning neural network image analysis of biglycan immuno-histochemical staining, showing reduced biglycan expression in cancer tissues. Indeed, it has been suggested that biglycan tissue expression datasets could be used to train and validate machine learning models in order to recognize, diagnose, and perform classification of breast cancer, utilizing biglycan stain identity [88]. On the other hand, an increased expression of other PGs, including the HSPG syndecan-4, was correlated with a worse survival in the subgroup of estrogen/progesterone-receptor negative and estrogen receptor-negative breast cancer patients. Notably, aberrant syndecan-4 expression in breast cancer was attributed to both transcriptional and post-transcriptional mechanisms [89]. These data suggest that PGs exert discrete tissue type-dependent effects in tumorigenesis. Biglycan’s expression and roles in cancer are summarized in Table 1.

Research into lumican in different cancer types also shows that its expression can be correlated with the disease’s progression. For example, lumican protein was found to be overexpressed in gastric cancer tissues compared to healthy tissues [90]. Furthermore, lumican expression was significantly correlated with the invasive potential in gastric cancer, suggesting that lumican is an independent prognostic factor [90]. In agreement with these earlier reports, subsequent studies utilizing R software and quantitative Real-Time PCR analysis, together with a comprehensive meta-analysis of gene expression profiles and gastric cancer patient’s clinical data from the Cancer Genome Atlas database, demonstrated substantial variation in lumican expression when comparing gastric cancer tissues and adjacent healthy tissues [91,92]. Notably, other PGs, including the cell membrane HSPG syndecan-4, are overexpressed in gastric cancer [93]. Indeed, syndecan-4 overexpression has been correlated with poor patient prognosis. Syndecan 4 is suggested to modulate gastric cancer cells’ invasion and motility [93]. Likewise, prospective studies must be performed to validate lumican as a prognostic marker in gastric cancer. Lumican expression in colon cancer was also correlated with lymph node metastasis, tumor invasion, and a lower survival rate [94]).

As far as breast cancer is concerned, lumican mRNA was present in the tumor stroma cells and associated with higher tumor grade, lower expression of estrogen receptors (ERs), and the patient’s younger age [95]. On the other hand, studies using breast cancer cell lines with discrete estrogen receptor (ER) expression demonstrated that lumican affects cancer cell functions and modulates the expression of ECM molecules in an ER-dependent manner [96]. Moreover, lumican is suggested to abrogate or even counter the epithelial-to-mesenchymal transition (EMT) of breast cancer cells [97]. Another type of cancer investigated was melanoma, in which lumican expression was identified in the dermis and peritumoral stroma, but not in melanoma cells [98]. Karamanou et al. demonstrated that lumican significantly reduced melanoma metastasis in the lungs in vivo and cell invasion in vitro [99]. In contrast, human melanoma cell lines expressed and secreted lumican, suggesting its possible involvement in the progression of malignant melanoma [100]. Lumican’s expression and roles in cancer are summarized in Table 2.

The above studies show a strong correlation between biglycan and lumican expression and cancer progression. The majority of these studies suggest that lumican and biglycan overexpression is negatively associated with cancer progression. Interestingly, some studies in specific tissues, such as breast tissue, indicate that these SLRPs exert an inhibitory role. Focusing on histological and functional aspects of discrete tissue could shed further light on contributions of biglycan and lumican.

## 5. SLRPs Modulate Cancer Cell Adhesion, Migration, and Invasion

Migration and adhesion are cell functions that are correlated with invasion and directly involved in embryo tissue development, tissue homeostasis, and regeneration. Invasion cell properties are also needed during the penetration of biological barriers that lead to metastasis [101]. During the epithelial-to-mesenchymal transition of cells in tumors, cells experience loss of intrinsic polarity, resulting in the loosening of cell-to-cell junctions. The latter process is supported by reorganizing the cell cytoskeleton and actin-based cell motility activation [102]. Cells that partially retain their polarity can form local adhesions and still attach to the ECM and perform cell body translocation [102]. Furthermore, cells with no polarity are amoeboid-like and can chemotactically move towards stimuli due to increased migration capacity and weakly attaching to the ECM [102].

### 5.1. Biglycan

Biglycan was described to modulate tumor cell migration and invasion capabilities. In the case of endometrial cancer, biglycan regulated cell invasion and migration cell capacity in vitro and experiments using a xenograft model [103]. A recent study showed that the sponging of miR-320a upregulates biglycan expression to support head and neck squamous cell carcinoma (HNSCC) progression by inducing EMT [104]. In this case, HNSCC cells exhibited increased migration and invasion, as well as enhanced expression of EMT markers, such as N-cadherin and vimentin.

**Figure 1:**
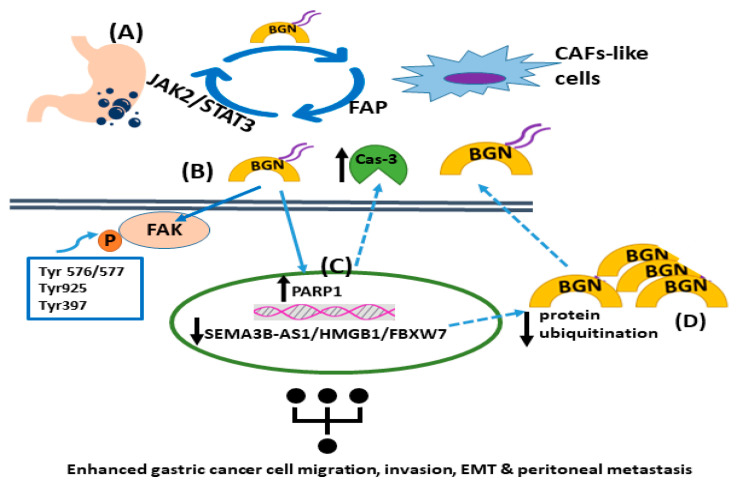
The roles of biglycan in gastric tumor carcinogenesis. (**A**) Gastric cancer cell-derived biglycan enhances the transformation of mesenchymal cells into CAF-like cells, which activates TLR signaling. In turn, CAF-like cells secrete fibroblast activation protein (FAP), promoting gastric cancer migration, invasion, and EMT. FAP initiates the JAK2/STAT3 signaling pathway in gastric cancer cells, increasing their biglycan expression. (**B**) Biglycan stimulates gastric cancer cell invasion and metastasis by activating FAK signaling via specific phosphorylation at Tyr576/577, Tyr925, and Tyr397. (**C**) Biglycan decreases PARP1 levels and caspase-3 cleavage with a concomitant upregulation of mesenchymal markers. (**D**) The SEMA3B-AS1/HMGB1/FBXW7 signaling axis is downregulated in gastric cancer cells, which facilitates the peritoneal metastasis of gastric cancer by decreasing biglycan protein ubiquitination and concomitant degradation, leading to higher biglycan levels.

In addition, biglycan, via in vitro and in vivo models of gastric cancer, was shown to increase tumor cell invasion and metastasis through activation of FAK signaling via specific phosphorylation at Tyr576/577, Tyr925, and Tyr397 [105]. Wu et al. recently presented a positive feedback loop that involved biglycan-, FAP-, and STAT3-mediated interactions between tumor cells and mesothelial cells that contribute to peritoneal metastasis of gastric cancer [106]. Specifically, this study identified an intricate loop through which gastric cancer cell-derived biglycan facilitates mesenchymal cell transformation to CAF-like cells, activating TLR signaling. In turn, CAF-like cells secrete fibroblast activation protein (FAP) to promote gastric cancer invasion, migration, and EMT. Moreover, FAP triggers the JAK2/STAT3 signaling pathway in gastric cancer cells, facilitating their biglycan expression to initiate the vicious circle of cancer progression [106]. In a separate study, the SEMA3B-AS1/HMGB1/FBXW7 signaling axis was shown to be decreased in gastric cancer cells, which enhanced the peritoneal metastasis of gastric cancer by modulating biglycan protein ubiquitination and concomitant degradation, resulting in higher biglycan levels [107]. Likewise, biglycan-deficient gastric cancer cells display attenuated migration compared to biglycan-expressing cells. Indeed, biglycan knockout gastric cancer cells have enhanced PARP1 levels and caspase-3 cleavage with a concomitant attenuation of mesenchymal marker expression. The in vitro and in vivo data were verified via patient biopsies [108]. These findings are supported by a study that showed that celastrol, which is a necroptosis inducer, triggered RIP1/RIP3/MLKL signaling pathways and attenuated pro-inflammatory cytokine release by targeting biglycan in gastric cancer cells [109]. The mechanisms of biglycan action in gastric cancer are presented in Figure 1.

Recently, biglycan was shown to enhance specific breast cancer stem cells’ (BCSC) migration and invasion. In this study, biglycan-deficient BCSCs exhibited decreased metabolism, reduced proportions, and attenuated ability to form tumor spheroids. Mechanistically, these cells demonstrated a decrease in NFκB transcription factor activity and downregulated phospho-IκB levels [110]. Moreover, ALDH^+^ and CD29^hi^ CD61^+^ BCSCs deficiency in biglycan were characterized by reduced metastatic ability. Manupati et al. suggest that biglycan could be a therapeutic target in BCSCs, making it helpful in developing novel strategies. Furthermore, biglycan was found to reduce HER-2/neu-transformed cells’ tumor properties, which were shown to be inversely correlated with PKC signaling [111].

Moreover, K-RAS-transformed colorectal cancer cells, which overexpress biglycan exhibit lower migratory properties [112]. These authors suggest that K-RAS-dependent malignancies could be treated via the upregulation of biglycan.

Datsis et al. investigated the role of biglycan in mesenchymal-originating tumors [113]. A co-operative mechanism of parathormone (1–34) and FGF-2 action was demonstrated using osteosarcoma cell lines to enhance biglycan expression and increase cell migration [113].

In contrast, this SLRP was determined to affect, in colorectal cancer, the desmoplastic reaction, inhibiting migration and invasion of these tumor cells in 2D and 3D coculture systems [114]. In cancer, the stroma tends to produce, in an unorganized fashion, fibrous tissue that consist mainly of collagen [115]. This response is named desmoplasia, and the fibrotic tissue is primarily synthesized by cancer-associated fibroblasts [115,116]. Another study on cell motility and biglycan in pancreatic cancer cells explains that the RAC1B/SMAD3/biglycan signaling axis attenuated migration [117]. These data demonstrate a complex pattern of biglycan actions, highlighting the need for more detailed studies.

### 5.2. Lumican

Cells’ 2D and 3D migration are facilitated by forming locomotory cytoplasmic (filopodia, pseudopodia, lamellipodia) or plasma membrane (invadopodia) protrusions that adhere to ECM components with the help of protein degradation molecules, like matrix metalloproteinases (MMPs) [118,119,120]. Invadopodia and lamellipodia formation were shown to be inhibited by lumican in prostate cells [121]. Experiments that plated prostate cancer cells on a lumican substrate resulted in the inhibition of lamellipodia formation via reduced rearrangement of ZO-1, keratin 8/18, integrin β1, and MT1-MMP. In addition, invadopodia inhibition was observed through disruption of α-smooth muscle actin, cortactin, and N-WASP [121]. Similarly, attenuation of invadopodia formation by lumican was shown in melanoma cells [122]. Furthermore, lumican was proven to affect the Snail-dependent MMP-14 action in melanoma cells; however, this process did not occur in the HT-29 colon adenocarcinoma cell model [123]. Furthermore, lumican reduced melanoma migration capacity via core protein binding to α2β1 integrin [124].

This SLRP modulates the expression of ECM molecules and reduces MMP-releasing invadopodia to inhibit melanoma lung metastasis in vivo and cell invasion, as Karamanou et al. (2021) recently showed. Also, lumican in breast cancer modulated cell morphology, while EMT decreased the expression of ECM regulators, like MMPs, and inhibited cell functions, such as migration and invasion [125].

In contrast, inhibition of lumican expression reduced bladder cancer cells’ migration capacity through MAPK signaling pathway activation [126]. In a separate study, lumican enhanced the adhesion of lung cancer cells to different ECM components and increased their in vitro cell migration, invasion, and osteogenic metastasis [127]. Furthermore, lumican overexpression increased podosome-like protrusions’ formation and migration in human colon cancer [128]. Experiments in an osteosarcoma cell model showed that lumican aided cell migration and chemotactic response to fibronectin, as well as facilitating the TGFβ2-dependent adhesion capacity of the same cells onto fibronectin [44,52,129]. Moreover, a FOX-3-dependent increase in lumican expression facilitated the migration of aggressive neuroblastoma cells [130].

The altered stroma ECM could significantly regulate growth, migration, and cancer spread [115]. Patients with lumican-positive stains of cancer stroma in pancreatic cancer presented shorter survival rates than those with lumican-negative stroma [131]. The different roles of lumican are summarized in Table 3.

Biglycan and lumican were proven to exert cancer-type specific roles in these cancer cell functions; thus, further investigation should aim to complete the detailed description of respective SLRPs’ mechanisms of action.

## 6. SLRPs Affect Tumor Cell Growth and Cell Cycle Regulation

One of the main steps involved in tumorigenesis is uncontrolled and sustainable cell proliferation. The deregulated function of cancer cells leads to the continuous release of growth-promoting signals, such as growth factor production that binds their tyrosine kinase activity receptors and activates their signaling, finally enhancing malignant transformation and tumor progression [132]. The interaction of SLRPs with vital signaling molecules on the cell surface or in the microenvironment is well-established [2]. Biglycan and lumican can positively or negatively affect malignant cell growth, depending on the type of cancer and their role in this particular tumor’s behavior.

### 6.1. Biglycan

**Figure 2:**
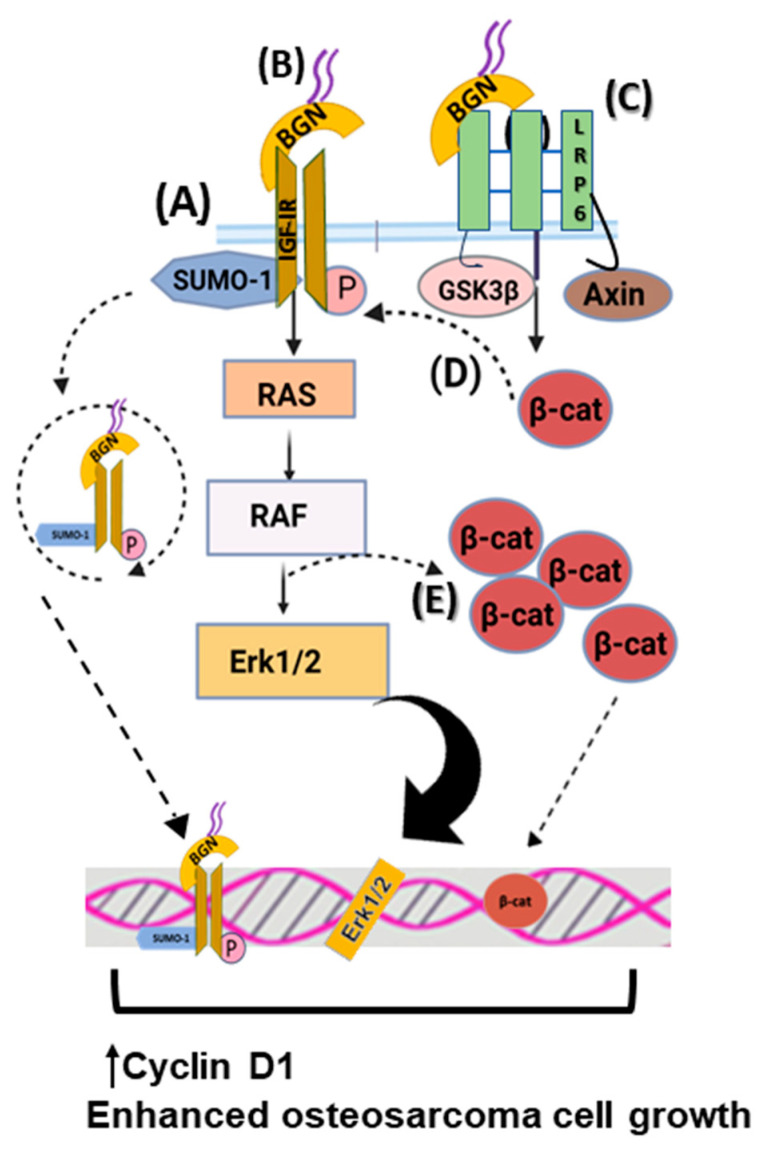
Biglycan upregulates osteosarcoma cell growth. (**A**) Biglycan binds to IGF-IR, enhancing its activation, subsequent sumoylation, and translocation to the nucleus. Nuclear IGF-IR regulates the transcription of target genes, including Cyclin D1, thus increasing osteosarcoma cell growth. (**B**) Upon biglycan binding to IGF-IR and its phosphorylation, downstream RAS/RAF/Erk_1/2_ signaling cascade is activated, resulting in transcriptional regulation that enhances osteosarcoma cell growth. (**C**) Biglycan binds to LRP6 and disrupts the formation of the β-catenin degradation complex, resulting in β-catenin nuclear translocation and transcriptional regulation that promotes osteosarcoma cell growth. (**D**) β-catenin co-localizes with IGF-IR, prolonging its activation and downstream signaling, which is correlated with increased osteosarcoma cell proliferation. (**E**) Activated Erk1/2 and β-catenin co-localize to facilitate β-catenin intracellular pool.

Biglycan is reported to significantly stimulate mesenchymal-derived tumor cell growth through complex regulating mechanisms. Thus, biglycan activates the insulin-like growth factor receptor I (IGF-IR) and Wnt/β-catenin signaling cascade, enhancing osteosarcoma growth. Biglycan-deficient cells show reduced activation of ERK1/2 and attenuated expression of Cyclin D1, which is a gene related to cell-cycle control [23]. Moreover, biglycan is shown to bind the IGF-IR receptor, enhancing its sumoylation and translocation to the nucleus. The nuclear IGF-IR has a transcriptional role, affecting the expression of target genes, including Cyclin D1 [25]. Moreover, computational studies reveal that biglycan changes the conformation of Wnt co-receptor low-density lipoprotein receptor-related protein 6 (LRP6) and promotes its ability to interact with signaling molecules. Biglycan binding to LRP6 disrupts the formation of the β-catenin degradation complex, resulting in β-catenin nuclear translocation and transcriptional upregulation of target genes, including Cyclin D1 [23,25]. Moreover, β-catenin was shown to co-localize with IGF-IR, prolonging its activation and downstream signaling. Previously, biglycan was shown to stimulate canonical Wnt signaling through binding to LRP6, resulting in enhanced bone fracture healing [133]. Collectively, these data suggest that by hijacking biglycan growth-promoting signaling, tumors can enhance their growth. The effects of biglycan in regulating osteosarcoma cell growth are depicted in Figure 2.

Notably, the LRP6/biglycan axis was, likewise, detected in glioblastoma cells, suggesting that it could also be relevant in advancing neural exoderm-derived malignancies [134]. Specifically, in a syngeneic glioblastoma mouse model, biglycan expression was increased in areas infiltrated with brain tumor-initiating cells. Treating brain tumor-initiating cells via exogenous biglycan or its overexpression increased their growth through biglycan binding to LRP6 and activation of Wnt/β-catenin signaling [134].

Biglycan, likewise, promotes the progression of epithelial-derived colorectal cancer. HCT116 biglycan-deficient colon cancer cells exhibit altered expression of cell-cycle regulator genes and lower proliferation rates, as well as cell-cycle arrest at the G0/G1 phase [135,136]. As hedgehog signaling, which results in biglycan overexpression, is overactivated in colon cancer, this process leads to enhanced tumor cell growth in colony formation and xenograft assays [137].

In addition to mainly growth-promoting effects, some studies show that biglycan inhibits cancer cell growth. For example, in pancreatic cancer, biglycan overexpression leads to G1 arrest and attenuates cell proliferation [138]. In addition, human urothelial carcinoma cells (J82 cells) that were deficient in biglycan exhibited enhanced proliferation and a decreased ability to form xenografts. Furthermore, treating J82 cells with recombinant biglycan in vitro strongly decreased their proliferation, confirming biglycan’s antiproliferative effect [86].

### 6.2. Lumican

It is well established that lumican controls tumor cells’ proliferation in a cancer-type-dependent manner. Thus, lumican’s positive correlation with gastric cancer is reported. CAFs in gastric cancer express lumican, which promotes the activation of the integrin β1-FAK signaling pathway, resulting in increased proliferation of cancer cells and tumor progression. Knockout of lumican reduces cell growth and metastasis in vivo [139]. Furthermore, lumican enhances cell proliferation in lung cancer cell lines, as lumican deficiency affects mitotic spindle and midbody formation, leading to chromosome mis-segregation and increased chromosome instability. Lumican-deficient H460 and A549 in non-small lung cancer cell lines exhibit a prolonged doubling time and stunted cell growth [140].

Regarding chondrosarcoma, lumican has a major role in cell proliferation mechanisms. A prior study demonstrated that lumican is the most abundantly expressed SLRP in HTB94 chondrosarcoma cells, and its deficiency is connected to reduced phosphorylation levels of IGF-IR and extracellular signal-regulated kinase ERK1/2 activation, which is a crucial signaling pathway for IGF-I-dependent HTB94 cell growth [26]. Lumican also modifies cancer response to chemotherapeutics [141]. A novel quinoline compound 91b1, which has strong anticancer effects in different cancer cell line models and in vivo, downregulates lumican mRNA levels, which are overexpressed in many cancers. Zhou et al. correlate lumican downregulation with suppressed cell proliferation and modulated cell cycle progression [142].

In osteosarcoma, lumican expression did not affect the growth of the aggressive MG63 cells, while attenuated non-aggressive Saos-2 cells grew through the TGF-β2 signaling cascade, affecting the bioavailability of Smad 2 activators [44,52]. Notably, in pancreatic ductal adenocarcinoma (PDAC) cancer, lumican, which is expressed by stromal cells, has an antiproliferative effect. Indeed, lumican’s expression attenuates the expression and activation levels of epithelial growth factor receptor (EGFR), leading to subsequent downregulation of Akt kinase activity and reduced tumor growth. Li et al. also demonstrated that PDAC cell treatment with exogenous lumican promotes cell-cycle arrest at G0/phase and cell-growth inhibition mediated via decreased ERK1/2 activation and increased p38 phosphorylation in PDAC cells [143,144]. Moreover, lumican inhibits tumor growth in melanoma mouse models by sensitizing the matrix microenvironment to ECM-targeted therapy [145]. These data suggest that stroma-derived lumican negatively affects cancer cell functions.

## 7. Biglycan Effects on Cancer-Associated Inflammation

The early detection of immune cells in cancer surroundings indicated that cancerogenesis is associated with inflammation [146].

Some of the pro-tumorigenic effects of biglycan are reflected in its participation in inflammatory and cellular immune responses [147]. Notably, biglycan affects both innate and adaptive immunity mechanisms [147,148]. Thus, the ECM-derived soluble form of biglycan acts as a danger signal by triggering an inflammatory response via the toll-like receptor (TLR)2/TLR4 in macrophages and dendritic cells [147]. According to molecular studies, the soluble protein acts as an ECM damage-associated molecular pattern (DAMP) that binds to innate immunity Toll-like receptors, i.e., TLR2 and TLR4, on the surface of macrophages [149]. Biglycan also attaches to the CD14 co-receptor via its protein core. This interaction is an obligatory step for a biglycan-dependent inflammatory signaling cascade via TLR2 and TLR4 [147,150], which releases cytokines and chemokines and recruits inflammatory cells [151]. Thus, in a model of renal inflammation, hepatocyte-derived biglycan induces leucocyte recruitment and infiltration of the renal parenchyma by the TLR adapter protein MyD88 [70].

The induction of sterile inflammation by biglycan through its interaction with TLR receptors on the surface of macrophages is followed by nuclear translocation of nuclear factor kappa-light-chain-enhancer of activated B cells (NF-κB), as well as the emergence of its transcriptional role and the activation of p38 and ERK signaling cascades. The following upregulated expression of pro-inflammatory cytokines, like tumor necrosis factor-α (TNF-α) and macrophage inflammatory protein-2 (MIP-2), triggers and regulates inflammation [69].

On the other hand, biglycan is negatively correlated with CD8+ T cell infiltration in triple-negative breast cancer, suggesting that it facilitates immunosuppressive mechanisms associated with adaptive immunity [148]. Moreover, gene expression data and corresponding clinical information from gastric cancer patients revealed that biglycan mRNA expression positively correlates with NK cell and macrophage infiltration, but negatively correlates with Th17 T cell enrichment [152]. Likewise, the higher expression of this SLRP in patients with head and neck squamous cell carcinoma is related to the less tumor-infiltrating CD4+ T, macrophages, and dendritic cells in these tumors [153]. Some studies have shown a discrete regulation of adaptive immune cell population by biglycan. Thus, biglycan is negatively correlated with B cell deposition, but has positive correlations with CD8+ and CD4+ T cells, as well as neutrophils, macrophages, and dendritic cells [73].

Moreover, a biglycan that was mediated and dependent on the TLR adapter protein TRIF cross-talk between innate and adaptive immunity was detected. Thus, mice deficient in TRIF had decreased infiltration of T-cells into the renal parenchyma [70]. Notably, biglycan through a TLR4/NF-B pathway was shown to contribute to the epigenetic silencing of Siglec-7 ligands. Since Siglec-7 is an immunosuppressive molecule, this case represented another example of a biglycan-dependent mechanism promoting carcinogenesis [85].

Biglycan has been correlated with the progression of blood cell cancers. Thus, in myelodysplastic syndromes (MDS) that progress to secondary acute myeloid leukemia (sAML), increased expression of biglycan was determined [154]. Indeed, all MDS and sAML patients’ biopsies and cell lines showed increased biglycan expression, mainly to the cell surface, compared to low expression of normal donor cells [154]. Enhanced biglycan was determined to mediate the inflammasome activity of CD34-CD33+TLR4+ cells in an MDS cell line. Moreover, biglycan expression was positively correlated with MUM1+ and CD8+ in bone marrow biopsies, whereas a negative correlation with CD33+TLR4+ cell infiltration was established. Increased biglycan expression was associated with decreased progression-free survival in MDS patients [154]. This study clearly shows biglycan inflammation-mediating abilities and the feasibility of targeted therapies in hematologic neoplasms.

Even though biglycan inflammatory activities are mostly correlated to protein core binding, the contribution of CS/DS chains has also been implicated. Thus, the CS/DS biglycan glycanation is suggested to contribute to the regulation of innate to antigen-specific adaptive immunity [155].

Therefore, a complex pattern of biglycan regulation of cancer-associated inflammation is emerging. Putatively, biglycan could promote the early steps of tumorigenesis by stimulating the inflammatory response, whereas in the later stages, it contributes to the generation of an immunosuppressive environment and tumor progression.

## 8. Biglycan Affects Tumor Angiogenesis

Notably, angiogenesis is a hallmark of cancer. Its role in tumor progression is to supply the tumor with the necessary nutrients and oxygen and enable malignant cells to metastasize through the circulation system [156]. A cancer is surrounded by stromal and immune cells, which form the tumor microenvironment and support tumor development by secreting molecules that activate endothelial cells. Under inflammatory conditions, vascular hyperpermeability allows inflammation mediators and immune response cells to infiltrate the site of malignancy. The vascular endothelial growth factor (VEGF) controls vascular permeability and participates in angiogenesis [157]. Among the several homodimeric glycoproteins comprising the VEGF family, the prototypical VEGF—VEGF-A—has been characterized as the strongest enhancer of vasculogenesis and angiogenesis [158]. In addition to increased vascular permeability, vasodilatation, and the recruitment of inflammatory cells, VEGF triggers the inhibition of apoptosis and increases cellular proliferation. The production of angiogenic signaling molecules leads to tumor vascularization, which is an essential step in tumor progression [142]. ECM activity is crucial to the distinct phases of the inflammation-associated angiogenic response [159].

In xenografts of colon tumors, biglycan overexpression activates the ERK signaling pathway and upregulates VEGF expression, leading to enhanced angiogenesis and tumor growth [136]. Furthermore, biglycan, through its binding to TLR2, TLR4, and CD14, mediates inflammation-dependent angiogenesis and affects interactions between the tumor parenchyma and stroma. Yamamoto et al. demonstrated that biglycan expression levels were higher in tumor endothelial cells (TECs) than in normal endothelial cells (NECs), and they affected cell migration and TECs’ cell morphology [160]. Maishi et al. proposed an epigenetic mechanism that regulates biglycan expression via TECs isolated from tumors with high metastatic potential. Hypomethylation in biglycan’s gene promoter leads to its overexpression and metastasis promotion [161]. Knockout of biglycan in the stroma of E0771 breast cancer-bearing mice led to inhibited lung metastasis, angiogenesis, and normalized tumor vasculature through downregulation of TNF-α/angiopoietin 2 signaling [162]. This result was correlated with enhanced chemotherapeutics drug delivery and efficiency [162].

In continuation, inhibition of biglycan expression in the TECs of renal cell carcinoma using a novel in vivo siRNA delivery system led to attenuated tumor growth. The blood vessels in the tumors that were deficient in biglycan exhibited smoother morphologies and had fewer sprouts, while TME factors, like fibrosis, were normalized [163].

Moreover, vessel formation is enhanced via this SLRP signaling through TLR2/TLR4 and NF-κB activation in gastric cancer. Its secretion and binding on endothelial cell receptors are connected to increased interaction between the transcription factor HIF-1 and the VEGF promoter and endothelial cell VEGF’s production, migration, and tube formation [164]. Downstream activation and mis-regulation of these signaling pathways enhances cancer progression by promoting angiogenesis [165]. Furthermore, a cohort study revealed higher biglycan expression from CAFs than normal cancer-adjacent fibroblasts (NAFs) in patients with triple-negative breast cancer. The higher expression levels of this SLPR demonstrate the extension of new blood vessels from the existing vessels, which is connected to poor prognostic outcomes and the formation of an immunosuppressive tumor microenvironment [148]. Likewise, the expression of biglycan-positive blood vessels was found to be increased in prostate cancer patients who were progressing to castration-resistant prostate cancer compared to those in the non-progressing group [82].

**Figure 3:**
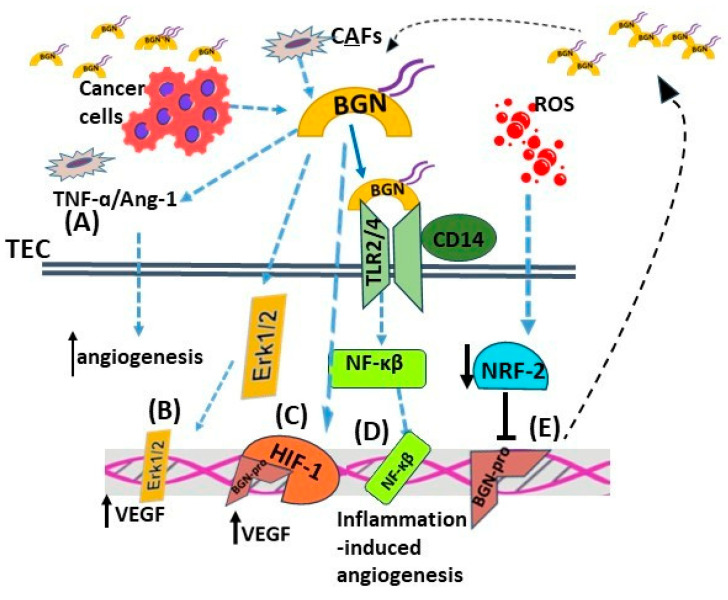
The effects of biglycan on tumor-associated angiogenesis. Biglycan secreted by cancer cells or CAFs can take the following actions: (**A**) modulate TNF-α/angiopietin-1 signaling to facilitate angiogenesis; (**B**) activate Erk1/2-dependent VEGF secretion, resulting in increased angiogenesis; (**C**) facilitate the interaction between the biglycan promoter (BGN-pro) and HIF-1, resulting in increased VEGF production; (**D**) biglycan, by binding to TLR2/4/CD14, enhances inflammation-dependent angiogenesis; (**E**) ROS inhibits the expression of a negative regulator of biglycan transcription NRF2 in TECs, the increased of which expression enhances their pro-angiogenic phenotype.

Notably, the reactive oxygen species (ROS) inhibited the expression of nuclear factor erythroid 2-related factor 2 (NRF2) in TECs. Since NRF-2 is a negative regulator of biglycan transcription, the levels of this SLRP were increased in TECs, inducing their pro-angiogenic phenotype [166]. The effects of biglycan on tumor-associated angiogenesis are summarized in Figure 3.

Collectively, these data suggest that biglycan inhibition can cause an antiangiogenic effect and be used as a potential target to control tumor-associated angiogenesis.

## 9. Biglycan and Lumican Affect Cell Death Mechanisms and Chemoresistance

Programmed cell death by apoptosis is a defense mechanism of cells against cancer development. Apoptosis is triggered in response to either cellular stress under physiological conditions or anticancer therapy. It is regulated via extracellular death-inducing or intracellular stress-related signals [132]. Autophagy is a catabolic mechanism that recycles cellular organelles under stress conditions to reuse them for biosynthesis and energy metabolism processes. The role of autophagy in cancer is dual, depending on the stage of tumor development [167]. Apoptosis and autophagy are distinct programs controlled by common signaling cascades that participate in cancer initiation and progression [168]. The involvement of SLRPs in the regulation of these processes is already known.

Substantial data reveal the role of biglycan in autophagy-dependent mechanisms. Biglycan increases the autophagosome formation and the autophagic flux in macrophages in a TLR4-CD44-dependent manner. The stimulation of this signaling pathway affects adhesion, migration, lymphocyte activation, and angiogenesis [169]. Biglycan-triggered autophagy enhances the anti-inflammatory M2 macrophage polarization, inflammation resolution, and tissue repair [170]. In neuroblastoma cells, biglycan attenuates autophagy-dependent AMPK–mTOR signaling and the intracellular levels of reactive oxygen species (ROS) to protect cells against nitric oxide (NO)-induced cytotoxicity [91]. Mintz et al. showed that biglycan is one of nine differentially expressed osteosarcoma genes found between responders and non-responders to chemotherapy [171]. A recent study reveals that biglycan decreases the expression of LC3, which is a well-studied autophagy marker, in two osteosarcoma cell lines. Biglycan-deficient cells treated with doxorubicin respond more effectively to this agent [29]. Fang et al. demonstrated that biglycan’s overexpression induces rapamycin resistance in WERI-Rb-1 retinoblastoma cells by activating the PI3K/Akt/NF-κB axis [172].

Moreover, in colon cancer, biglycan overexpression in stem cells was found to be associated with the activation of the NF-κB pathway. It decreased the expression of pro-apoptotic markers, promoting colon cancer cell chemoresistance [71]. Biglycan is shown to be overexpressed in gastric cancer tissue, and it negatively affects patients’ clinical outcomes. In vitro and in vivo experiments indicated that biglycan knockout gastric cancer cells exhibit decreased cell survival, migration, and angiogenic potential compared to cells that express this SLRP. This mechanism implicated the regulation of mesenchymal markers and PARP-1 expression levels, as well as caspase-3 cleavage. Exogenous treatment with biglycan restores their survival, clonogenicity, and migration [108]. Biglycan may affect tumor response to chemotherapy through its ability to control autophagic and apoptotic mechanisms.

Hypoxia induces autophagy in pancreatic cancer stellate cells through a lumican-dependent mechanism. Secreted lumican reduced EGFR expression, and phosphorylation inhibited Akt kinase activity and, subsequently, attenuated the expression of hypoxia-inducible factor-1α(HIF-1α), thus altering glucose consumption, lactate production, intracellular ATP, and cell apoptosis [143]. Hypoxia-induced autophagy in pancreatic cancer stellate cells decreases lumican expression post-transcriptionally by regulating a vital protein synthesis pathway. AMPK inhibition prevents hypoxia-reduced phosphorylation of the mTOR/p70S6K/4EBP signaling pathway, altering protein degradation and synthesis inhibition. Lumican secretion was strongly downregulated in PDAC [173]. In addition, lumican was found to regulate the apoptosis of corneal and embryonic fibroblasts through binding to the soluble Fas ligand, facilitating its presentation to Fas. Regulation of cyclin activation by p21 in a p53-dependent manner led to anti-proliferating effects. Without the presence of lumican, Fas–Fas ligand interaction is inefficient [174].

Regarding apoptosis, B16F1 mouse melanoma cells transfected to overexpress lumican present an initiation and/or increase in apoptosis [175]. Accordingly, Brézillon et al. recently demonstrated that a lumican-derived peptide (L9Mc) increased cell apoptosis of B16F1 cells in a mouse model of primary melanoma tumors [176]. Another study indicated that downregulation of lumican expression by bone marrow mesenchymal stem cells (BM-MSC) led to decreased apoptosis and chemosensitivity to agents such as VP-16 in human pre-B cell leukemia Nalm-6 cell line [177]. In PDAC models, chemotherapeutic agents promote autophagosome formation and enhance LC3 expression through the ROS-mediated AMPK signaling pathway. These drugs increased lumican secretion, decreasing AMPK activity and, finally, affecting the extent of response by inhibiting autophagy in in vitro and in vivo PDAC models [178]. Effects of biglycan and lumican on hey cell cancer function are summarized in Figure 2.

## 10. SLRPs as Therapy Targets

Given all of the different actions of biglycan and lumican in cancer cell function and tumor development described in this review, these SLRPs are considered candidate targets of anticancer therapies. However, to date, there are few experimental studies that consider the therapeutic roles of these molecules. One example involves using celastrol in gastric cancer cells [109]. A triterpene component of Chinese medicine, i.e., celastrol, activated receptor-interacting proteins 1 and 3 (RIP1 and RIP3) and, subsequently, facilitated the translation of mixed-lineage kinase domain-like protein from the cytoplasm to the plasma membrane in a biglycan-dependent manner [109]. Furthermore, celastrol decreased the secretion of pro-inflammatory cytokines TNF-α and IL-8 in HGC27 and AGS gastric cancer cells, which was reversed via overexpression of biglycan [109].

In a separate study, biglycan-dependent promotion of tumor endothelial cells (TECs) was targeted. Within this study’s scope, siRNA against biglycan was loaded onto a multifunctional envelope-type nano device (MEND) that contained a unique pH-sensitive cationic lipid. Cyclo(Arg-Gly-Asp-D-Phe-Lys), which is a ligand of αV β3 integrin highly expressed in TECs, was incorporated into MEND. The injection of encapsulated siBGN into MEND in nude mice carrying human renal carcinoma xenografts inhibited tumor growth [163]. In a correlated study, biglycan TEC levels were shown to be directly correlated with pre-operative biglycan serum levels in lung patients. Measuring biglycan serum levels in healthy subjects and lung cancer patients showed that they were lower in healthy controls and patients with less invasive adenocarcinoma [179]. Thus, Morimoto et al. suggested that low biglycan levels in pre-operative serum in lung cancer patients could identify common malignancies and allowed limited resection, which was beneficial for patients with low malignancy [179].

Likewise, the downregulation of stromal biglycan in E0771 tumor-bearing mice attenuated lung metastasis, decreased tumor angiogenesis, suppressed fibrosis, and increased CD8+ T cell infiltration. Moreover, the delivery of a chemotherapy drug and its efficacy were enhanced in vivo, suggesting that targeted downregulation of specific biglycan expression can form a novel anticancer strategy [162].

Biglycan has been reported to affect immune cell signaling through TLR2/4 [70,151,180]. Experimental data that support the latter method indicate that activation of TLR2/TLR4 downstream signaling can enhance tumor- and stromal-cell secretion of inflammation-associated cytokines, thus modulating tumor development [181,182,183]. In a pan-cancer study, high biglycan tissue expression was correlated with a poorer therapy response than low biglycan tissue expression and a decreased infiltration of CD8+ T cells, suggesting its potential for utilization as a prognosis/therapy marker [84].

As far as lumican is concerned, a study suggested that its ability to inhibit Snail activity, which is a significant trigger of epithelial-to-mesenchymal transition, may be used as a base for anticancer therapy [184]. Furthermore, a recent study showed that a quinoline derivative, i.e., compound 91b1, mediates its significant anticancer effects by downregulating the lumican gene [142].

Studies that focused on the possible beneficial role of biglycan and lumican in the fight against carcinogenesis are shown to be necessary.

## 11. Conclusions

The present review discusses the significant roles attributed, to date, to biglycan and lumican in cancer development. These SLRPs are involved in all vital functions of a cancer cell: proliferation, migration, invasion, regulation of immune action, and angiogenesis. Biglycan’s expression has been shown to mostly positively correlate with cancer development. In addition, biglycan was demonstrated to promote inflammation and angiogenesis in cancer progression. Notably, in osteosarcoma, biglycan could predict chemotherapy resistance. Lumican’s role has been shown to be both anti- and pro-carcinogenic, depending on the tumor’s origin and cancer stage. Moreover, evidence shows that lumican can predict the patient’s response to radiotherapy and regulate chemotherapy. Once again, considering the immense variety of the glycocode, greater focus on deciphering SLRPs’ glycosylation patterns and their correlation with respective signaling abilities is needed [185]. The tumor microenvironment that consisted of different cell types and signaling mediators was shown to be rich in ECM molecules that modulate both stroma and cancer cell functions. Therefore, the characterization of key ECM molecules in tumor progression is vital. Based on ongoing studies, SLRPs, biglycan, and lumican can be included as targets/biomarkers for use in future cancer therapy strategies.

## Figures and Tables

**Table 1:** Biglycan’s roles in cancer.

Role	References
Biglycan, which is attached to the matrix, is cleaved by proteases and released under pathological conditions.	[21,69]
Activated macrophages can produce de novo biglycan.	[70]
Overexpression of biglycan mRNA has been detected in the vast majority of cancer tissues and 28 cancer types using the Oncomine database.	[22]
Biglycan is overexpressed in human endometrial cancer, both in the parenchyma and mesenchyme compartments.	[71]
Biglycan’s gene expression was correlated with gastric cancer metastases.	[72]
Biglycan facilitates the incidence of lung adenocarcinoma through ECM–receptor interaction.	[75]
Biglycan was identified as a metastasis-specific biomarker in human colon cancer using integrated analysis.	[80]
Overexpressed biglycan was strongly correlated with prostate cancer development.	[81]
Biglycan expressed in the cancerous prostate stroma was suggested as a prognostic factor.	[82]
Bladder cancer patients displaying high biglycan expression exhibited reduced tumor cell growth and enhanced ten-year survival in patients.	[86]
High biglycan expression in oral squamous cell carcinoma was correlated with poor overall survival, as well as tumor-specific survival.	[74]
Biglycan protein expression was reduced in breast cancer tissue compared to normal tissue.	[87]
Single-cell RNA sequencing pan-cancer cohorts showed that enhanced biglycan production by CAF was negatively correlated with patient survival and response to therapy.	[84]

**Table 2:** Lumican’s roles in cancer.

**Role**	**References**
Lumican protein was overexpressed in gastric cancer tissues compared to healthy tissues. Lumican is suggested to be an independent prognostic factor, which needs to be verified in prospective studies.	[90,91,92]
Lumican mRNA present in the tumor stroma of breast cancer was correlated with higher tumor grade, lower expression of estrogen receptors (ERs), and the patient’s younger age.	[95]
In breast cancer cell lines, lumican affected cancer cell functions and modulated the expression of ECM molecules, abrogating EMT.	[97]
In colon cancer, lumican expression was associated with metastasis in lymph nodes, tumor invasion, and a lower survival rate.	[94]
In melanoma, lumican was expressed in the dermis and peritumoral stroma, but not in melanoma cells.	[98]
Lumican significantly reduced melanoma lung metastasis in vivo and cell invasion in vitro.	[99]
Lumican is expressed and secreted by some melanoma cell lines, suggesting its possible involvement in the progression of the disease.	[100]

**Role**

**References**

**Table 3:** Lumican’s actions in cancer adhesion, migration, and invasion.

Lumican Action	Type of Cancer	References
Inhibition of invadopodia and lamellipodia formation.	Prostate	[121]
Attenuation of invadopodia formation.	Melanoma	[122]
Regulation of Snail-dependent MMP-14 action.	Melanoma	[123]
Reduction in migration capacity via core protein binding to α2β1 integrin.	Melanoma	[124]
Modulation of ECM molecule expression and reduction in MMP-releasing invadopodia.	Lung	[99]
Modulation of cell morphology and EMT, reduction in ECM regulators, like MMPs, and inhibition of migration and invasion.	Breast	[125]
Inhibition of lumican reduced migration via the MAPK signaling pathway.	Bladder	[126]
Induction of adhesion, cell migration, invasion, and osteogenic metastasis.	Lung	[127]
Increased formation and migration of podosome-like protrusions.	Colon	[128]
Enhancement of cell migration, chemotactic response to fibronectin, and TGFβ2-dependent adhesion to fibronectin.	Osteosarcoma	[44,52,129]
Enhanced migration through a FOX-3-dependent increase in lumican expression.	Neuroblastoma	[130]
